# Effect of Mediterranean diet on body mass index and fatigue severity in patients with multiple sclerosis: A systematic review and meta-analysis of clinical trials^☆^

**DOI:** 10.1016/j.heliyon.2024.e37705

**Published:** 2024-09-14

**Authors:** Amir Reza Moravejolahkami, Mehdi Shakibaei, Manoj Sharma, Masoud Mohammadnezhad, Sri Lakshmi Sravani Devarakonda

**Affiliations:** a Department of Clinical Nutrition, School of Nutrition & Food Science, Isfahan University of Medical Sciences, Isfahan, Iran; b Institute of Anatomy, Faculty of Medicine, Musculoskeletal & Tumor Biology Research Group, Ludwig-Maximilians-University Munich, Munich, Germany; c Department of Social & Behavioral Health, School of Public Health, University of Nevada, Las Vegas, NV 89119, USA; d Department of Internal Medicine, Kirk Kerkorian School of Medicine, University of Nevada, Las Vegas, NV 89119, USA; e Faculty of Health, Education and Life Sciences, Birmingham City University, Birmingham, England; f Nutrition Program, Hunter College, City University of New York, New York, NY 10065, USA

**Keywords:** Mediterranean diet, Multiple sclerosis, Fatigue, Meta‐analysis, Systematic review

## Abstract

**Background:**

Recent studies imply that Mediterranean Diet (MeD) may play an important role in the management of Multiple Sclerosis (MS). This meta-analysis aimed to evaluate the effectiveness of MeD in addressing MS-related complications.

**Methods:**

A thorough search was performed in MEDLINE (PubMed), Scopus, EMBASE, ScienceDirect, Google Scholar, Web of Science, and the Central Cochrane Library, covering trials published until September 2023. The quantitative data were synthesized using random effect models through STATA_14_.

**Results:**

After analyzing 228 entries, we found five Randomized Controlled Trials (RCTs) with a total of 540 participants, who had an average disease duration of 8.5 years. The combined effect size revealed a decrease in Body Mass Index (BMI) (Weighted Mean Difference [WMD] = −0.88 kg/m^2^; 95 % Confidence Interval [CI] = −1.68, −0.08; P = 0.030). There was also a non-significant marginal improvement in fatigue severity (WMD = −8.29; 95 % CI = −16.74, 0.16; P = 0.054).

**Conclusion:**

Adherence to MeD may improve BMI and fatigue severity in MS patients. Further RCTs are needed to confirm the current results.

## 1 Introduction

Multiple Sclerosis (MS) is an inflammatory autoimmune disease that affects the central nervous system, particularly the brain and spinal cord. While the exact cause of MS is still unknown, research suggests that a combination of genetic and environmental factors contributes to development and progression [1].

One area of interest in MS research is the role of diet in managing the condition [2]. The Mediterranean Diet (MeD), characterized by high consumption of fruits, vegetables, whole grains, legumes, nuts, seeds, and olive oil, along with moderate intake of fish, poultry and dairy which has been associated with various health benefits [3]. Some researches considered the modified versions of MeD, mainly by removing wine intake, replacing fish and poultry with plant-based protein sources like legumes, tofu, tempeh, and using lactose-free dairy products or plant-based alternatives like almond milk or coconut yogurt if lactose intolerant [4,5].

Studies have highlighted several mechanisms through which the MeD may exert beneficial effects on MS patients [6]. The anti-inflammatory properties of MeD, derived from high consumption of fruits, vegetables, fish, and olive oil, may help reduce inflammation in the body, a key factor in the development and progression of MS [7]. Additionally, the antioxidant content, sourced from fruits, vegetables, and olive oil, can protect against oxidative stress, which has been implicated in MS pathogenesis [8]. Furthermore, emerging research suggests that MeD promotes a diverse and beneficial gut microbiota composition [9]. This modulation of the gut microbiome can influence immune function and inflammation, potentially ameliorating MS symptoms [10]. Overall, the MeD shows promise in managing MS by targeting inflammation, oxidative stress, and the gut microbiome, providing a holistic approach to improve the well-being of MS patients [11].

A recently published systematic review and meta-analysis encompassing eight Randomized Controlled Trials (RCTs) proposed dietary intervention as a potential approach for managing MS. However, the study did not conduct a sub-group analysis specifically focusing on the MeD [12]. Therefore, in this present review, we conducted a comprehensive reanalysis of relevant RCTs to determine the effectiveness of the MeD in MS patients, specifically concerning fatigue severity and Body Mass Index (BMI).

## 2 Materials & methods

### 2.1 Protocol validation

The research protocol was formulated in adherence to the guidelines stipulated by the Preferred Reporting Items for Systematic Reviews and Meta-Analyses (PRISMA) statement [13], as detailed in Supplementary Table 1. Furthermore, this systematic review was duly registered in the International Prospective Register for Systematic Reviews (PROSPERO) database under registration number CRD42022368118, with the registration date being October 30, 2022.

### 2.2 Literature search

Two researchers conducted independent searches on the specified electronic databases up until March 2024, detailed in Supplementary Table 2. The databases included the ISI Web of Science, Embase, Cochrane Central Register of Controlled Trials (CENTRAL), Google Scholar, ScienceDirect, MEDLINE (PubMed), and Scopus.

The search strategy encompassed both MeSH and non-MeSH keywords to ensure a comprehensive exploration: (("Mediterranean Diet" OR "MeD" OR "Diet" OR "Plant-based Diet" OR "Healthy Diet" OR "Mediterranean-like Diet") AND ("Relapsing-Remitting Multiple Sclerosis" OR "Multiple Sclerosis" OR "RRMS" OR "MS") AND ("Body Mass Index" OR "Fatigue" OR "Modified Fatigue Impact Scale" OR "MFIS" OR "BMI") AND ("Intervention" OR "Intervention Study" OR "Intervention Studies" OR "Controlled trial" OR "Randomised" OR "Randomized controlled trial" OR "Randomized clinical trial" OR "Randomized clinical trial" OR "RCT" OR "Non-Randomized Controlled Trials" OR "Clinical Trials" OR "Clinical Trial" OR "Trial" OR "Trials" OR "Non-Randomized Controlled Trials" OR "Cross-Over study" OR "Cross-Over trial" OR "Cross Over trial" OR "Cross Over study" OR "Double-Blind Method" OR "Double-Blind" OR "Double-Blind trial" OR "Double-Blind study")). Additionally, a thorough examination of the references list of the included trials was conducted to identify any supplementary pertinent papers for inclusion in the study.

### 2.3 Study and participants’ selection criteria

The language criterion for the included trials was restricted to English. Studies with single, double, or triple-blind designs, employing either parallel or cross-over methodologies and featuring a minimum of two arms, were chosen for evaluation. These studies assessed the impact of the MeD on BMI and fatigue severity in MS patients aged between 18 and 60 years, with accompanying control groups. The assessment process involved one author evaluating the relevance of articles and abstracts for potential inclusion. Subsequently, two authors independently scrutinized the full text of non-duplicated articles. Any discrepancies between the two reviewers were resolved by a third independent reviewer.

### 2.4 Data extraction

The extraction of data was conducted by a single author. The extracted data encompassed the following variables: (1) study first author, (2) publication year and study location, (3) study design and duration, (4) baseline characteristics of the samples including age, gender, disease duration, and Extended Disability Status Scale (EDSS), (5) details regarding the intervention and control groups, and (6) the main measured parameters such as BMI and Modified Fatigue Impact Scale (MFIS). MFIS is a validated instrument designed to assess the impact of fatigue on daily functioning in individuals with multiple sclerosis (MS). Comprising 21 items, the MFIS evaluates three domains: physical, cognitive, and psychosocial functioning. Respondents rate the frequency of fatigue-related experiences on a 5-point Likert scale, ranging from 0 (never) to 4 (almost always), with higher total scores indicating greater fatigue impact [14]. BMI is also a widely used metric for assessing body weight relative to height, calculated by dividing an individual's weight in kilograms by the square of their height in meters (kg/m^2^) [15]. In instances where data were ambiguous or incomplete, the data analyst reached out to the corresponding authors via email to obtain supplementary information.

### 2.5 Risk of bias (quality) assessment

The quality assessment of the included trials was conducted by two researchers using the Cochrane Risk-of-Bias (RoB) tool (version 5.0) [16]. In cases of discrepancies between the two assessors, a third assessor intervened to resolve any disagreements. Key biases evaluated included: (a) allocation concealment (selection bias), (c) blinding of participants and personnel (performance bias), (b) random sequence generation (selection bias), (e) incomplete outcome data (attrition bias), (d) blinding of outcome assessment (detection bias), and (f) selective reporting (reporting bias). The final determination of bias level was categorized as either “low risk,” “high risk,” or “some concerns” of bias.

### 2.6 Statistical analysis

The meta-analysis was conducted using STATA software version 14 (Stata Corp LP, College Station, TX, USA). Data were inputted into STATA as Mean difference with Standard Deviation (m±SD). The random-effects model was applied to calculate Weighted Mean Differences (WMDs) with 95 % Confidence Intervals (CIs). In cases where only the Standard Error of the Mean (SEM) was available, SD was calculated using the formula:

*SD = SEM* × *square root of n (n*; *the number of subjects)* [17].

When trial results were presented in median/Interquartile Range (IQR) or 95 % CI, mean and SD values were estimated following the method established by Hozo et al. [18]. The assessment of between-study heterogeneity was carried out using the I-square (I^2^) test. Sensitivity analysis was performed to evaluate the influence of removing a single study on the overall outcomes. Begg's rank correlation and Egger's weighted regression tests were employed to assess potential publication bias. A significance level of P < 0.05 was considered statistically significant.

## 3 Results

### 3.1 Search details

**Fig. 1:**
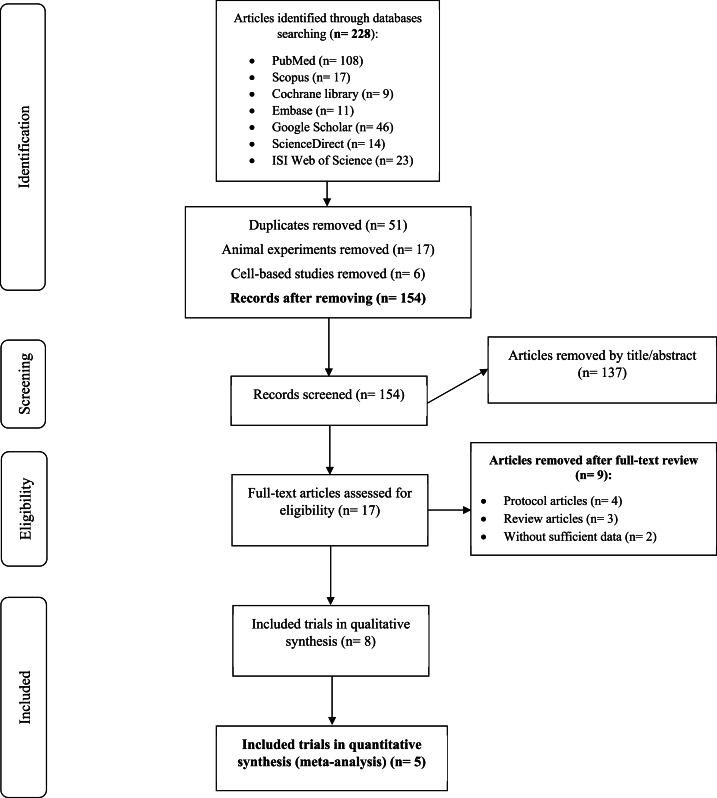
Flow diagram describing the included and excluded studies.

The initial literature review identified a total of 228 records, of which 74 were excluded due to duplication (51 studies), animal experiments (17 studies), and cell-based studies (6 studies). Following the evaluation of titles and abstracts, an additional 137 papers were excluded. Ultimately, five Randomized Controlled Trials (RCTs) comprising 540 patient records and published between 2020 and 2022 were selected for quantitative analysis [3,6,[19], [20], [21]] (Fig. 1).

### 3.2 Study characteristics

#### 3.2.1 Meta-analyzed studies

The key data extracted from the included Randomized Controlled Trials (RCTs) are summarized in Table 1 [3,6,[19], [20], [21]]. A total of 540 participants (270 in the intervention group, 270 in the control group) with an average age of 37.2 years and a mean disease duration of 8.4 years was identified; however, due to an average dropout rate of 19 % across the selected RCTs, data from 436 participants were reanalyzed. All RCTs adhered to a two-arm parallel design, encompassing both genders (with a female-to-male ratio of 4.5), with the exception of the study by Papandreou et al. [20], which exclusively enrolled female participants. Cultural considerations led to the exclusion of wine and pork meat intake in three studies due to limitations within the target populations [3,6,19]. The duration of the study follow-ups ranged from 12 to 52 weeks.

**Table 1:** Characteristic of randomized controlled trials that evaluated the effect of Mediterranean diet on Multiple Sclerosis (MS).

First author (publication year)	Country	Primary sample size In/Co Male/Female	Analyzed sample size In/Co Male/Female	Target population	Disease duration M (SD)	EDSS at base	Age (M)	Study design Duration	Intervention	Control	Main measured markers
Sand (2019)^a^	United States	18/18 Only female	15/15 Only femSale	RRMS SPMS PPMS	4.8	2.0	43.0	R, PC Parallel 24 wks	modified Mediterranean diet (limit salt intake to 2 g/day)	Usual diet (participation in educational (non-interventional) seminars on MS)	EDSS, MSFC, MSIS-29, MSQOL, MACFIMS cognitive battery, NFI-MS
Moravejolahkami (2020)^a^	Iran	90/90 40/140	68/79 25/122	RRMS	8.7 (6.3)	1.8 (0.8)	39.3	R, SB, PC Parallel 24 wks	Mediterranean diet (wine/pork meat-free)	Usual diet (traditional Iranian diet)	Dietary intake of macro&micro nutrients, MSQOL, VAFS, FSS
Moravejolahkami (2020)^a^	Iran	5 Only female	5 Only female	SPMS	9.0	4.1	34.6	Case series 52 wks	Mediterranean diet and synbiotics supplementation	ND	EDSS, FSS, MSQOL
Razeghi-Jahromi (2020)	Iran	40/40 NR	34/38 8/64	RRMS	8.0 (5.0)	2.3 (1.1)	34	R, SB, PC Parallel 52 wks	Mediterranean diet (wine-free)	Usual diet	MFIS, MACFIMS, BMI
Mirashrafi (2021)	Iran	90/90 40/140	68/79 25/122	RRMS	8.7 (6.3)	1.8 (0.8)	39.3	R, SB, PC Parallel 24 wks	Mediterranean diet (wine/pork meat-free)	Usual diet (traditional Iranian diet)	Energy intake, dietary intake of macro&micro nutrients, weight, BMI, relapse rate, percent body fat, hs-CRP
Ovcharova (2022)	Bulgaria	30/30 NR	17/13 13/17	RRMS	NR	3.5 (1.4)	43.9	open-label prospective nonrandomized 12 wks	Adapted Mediterranean diet	Regular diet with EPA/DHA food supplementation	BMI, FSMC, MFIS, EDSS, Chol, LDL, HDL, TG, CRP, IL17A, EPA, DHA, Fat, Visceral fat, Metabolic rate
Papandreou (2022)	Greece	20/20 Only female	20/20 Only female	RRMS	NR	0.87 (1.2)	29.5	R, DB, PC Pilot Parallel 12 wks	Mediterranean diet	General nutritional and physical activity advice	Weight, BMI, FFM, Glutamine, Chol, HDL, LDL, TG, CRP, Vitamin 1,25(OH)2D, METs-min/week, Mediterranean Diet Score, HADS, Dietary intake of fat, fiber, saturated fat, and MUFA
Bohlouli (2022)	Iran	90/90 40/140	68/79 25/122	RRMS	8.7 (6.3)	1.8 (0.8)	39.3	R, SB, PC Parallel 24 wks	Mediterranean diet (wine/pork meat-free)	Usual diet (traditional Iranian diet)	Energy intake, dietary intake of macro&micro nutrients, MFIS, EDSS

**Functional abbreviations:** In, intervention group; Co, control group; M, mean; SD, standard deviation; RS/DBPC, randomized single or double blind placebo control trial; wks, weeks; NR, not reported; ND, not defined.

**Study outcome abbreviations:** MACFIMS, Minimal Assessment of Cognitive Function in MS; MFIS, modified fatigue impact scale; FSMC, Fatigue Scale for Motor and Cognitive Functions; HADS, Hospital Anxiety and Depression Scale; MSQOL, multiple sclerosis quality of life-54; FSS, fatigue severity scale; VAFS, visual analogue fatigue scale; MSFC, Multiple Sclerosis Functional Composite; NFI-MS, Neurological Fatigue Index-MS; MSIS-29, Multiple Sclerosis Impact Scale-29; hs-CRP, high sensitive C-reactive protein; BMI, Body Mass Index; TG, Triglycerides; Chol, cholesterol; LDL, low density lipoprotein; HDL, high density lipoprotein; IL interleukin; EPA, eicosapentaenoic acid; DHA, docosahexaenoic acid; MUFA, monounsaturated fatty acid; FFM, free fat mass.

a Only systematically reviewed studies. The measured outcomes of three studies were different from those were specified in the selection criteria.

#### 3.2.2 Only systematically reviewed studies

The attributes of three studies are outlined in Table 1, with the publication dates spanning 2019 and 2020, from Iran and the USA [7,22,23]. It is worth noting that two Randomized Controlled Trials (RCTs) lacked matched target variables suitable for meta-analysis [22,23], while one study was a case-series [7]; therefore, it was impossible to include them in quantitative analysis. Moravejolahkami et al. [7] incorporated a co-intervention involving "synbiotics supplementation" in their study. Similarly, Sand et al. [23] implemented a modified version of the MeD with restricted salt intake (not exceeding 2 g per day). The duration of the study follow-ups ranged from 24 to 52 weeks.

### 3.3 Risk of bias (quality) assessment

**Fig. 2:**
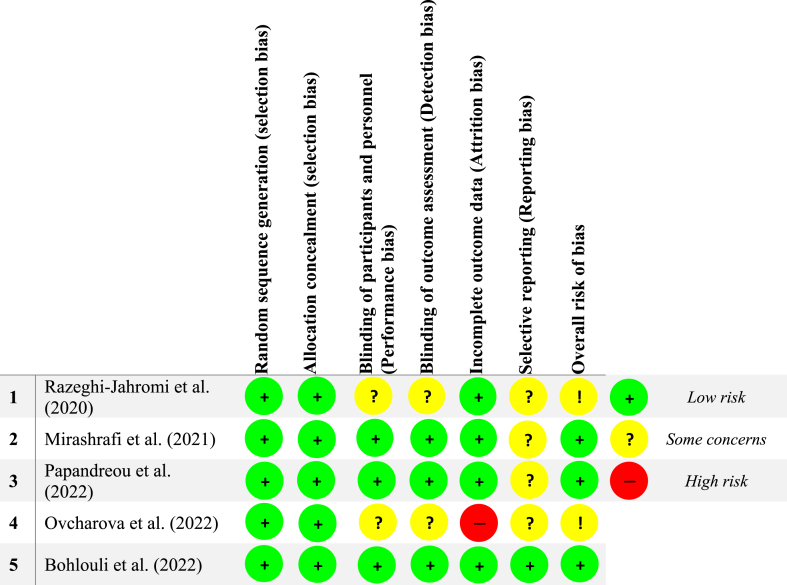
Risk of bias assessment for the meta-analyzed RCTs.

Three RCTs deemed to have a low risk of bias [3,6,20]; however, there were certain concerns regarding the risk of bias in two studies [19,21]. Notably, the risk of bias was ascertained to be low for random sequence generation and allocation concealment, which mitigates selection biases. However, uncertainties arose in relation to performance bias [19,21], detection bias [19,21], and reporting bias [6,[19], [20], [21]]. One study [21] exhibited a high risk of attrition bias. More details on quality assessment are shown in Fig. 2.

### 3.4 Results of meta-analysis

#### 3.4.1 MeD and BMI

**Fig. 3:**
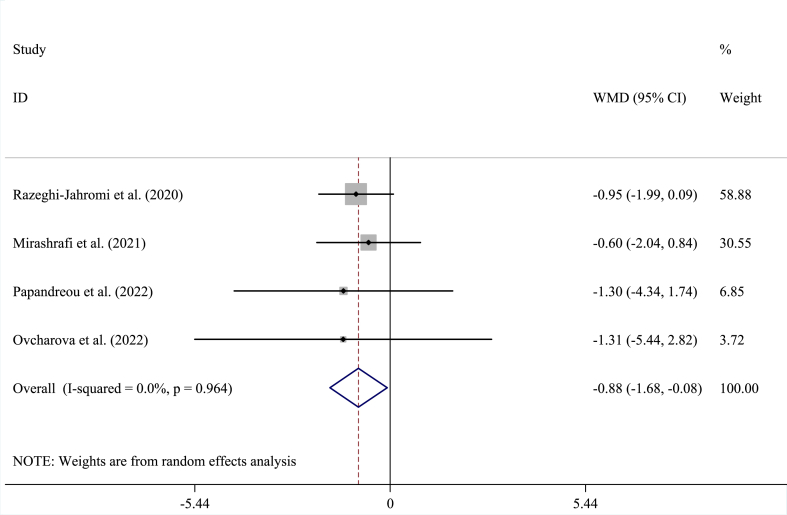
This plot depicts the results of randomized controlled trials assessing the efficacy of the Mediterranean diet on Body Mass Index (BMI) in individuals diagnosed with Multiple Sclerosis (MS). Each square's size corresponds to the inverse of the Weighted Mean Difference (WMD) variance. The horizontal lines represent the 95 % Confidence Intervals (CIs). The pooled effect size indicates a reduction in BMI subsequent to the Mediterranean diet intervention, with a statistically significant result (P = 0.030).

Within the pooled analysis encompassing four studies involving 289 participants (intervention: 139; control: 150) [6,[19], [20], [21]], effect of MeD on BMI (WMD = −0.88 kg/m^2^; 95 % CI = −1.68, −0.08; P < 0.030) was statistically signiﬁcant devoid of heterogeneity (I^2^ = 0.0 %; P = 0.964) (Fig. 3).

#### 3.4.2 MeD and MFIS

**Fig. 4:**
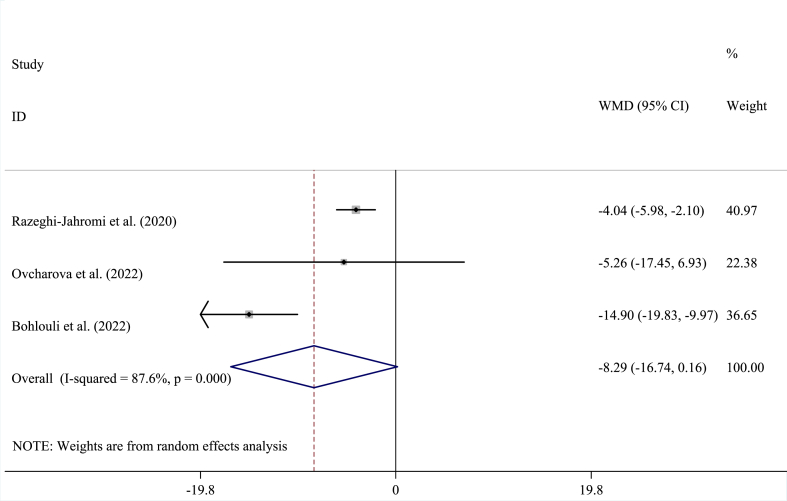
This plot depicts the results of randomized controlled trials assessing the efficacy of the Mediterranean diet on Modified Fatigue Impact Scale (MFIS) in individuals diagnosed with Multiple Sclerosis (MS). Each square's size corresponds to the inverse of the Weighted Mean Difference (WMD) variance. The horizontal lines represent the 95 % Confidence Intervals (CIs). The pooled effect size showed that Mediterranean diet marginally reduces fatigue severity in a NON-significant manner (P = 0.054).

While a decreasing trend in fatigue severity was observed, the pooled estimate did not indicate a significant enhancement in MFIS scores among patients adhering to the MeD (WMD = −8.29; 95 % CI = −16.74, 0.16; P = 0.054), as analyzed across three RCTs involving 249 participants [3,19,21]. Furthermore, there was identified study heterogeneity (I^2^ = 87.6 %, P < 0.001), as depicted in Fig. 4.

### 3.5 Sensitivity analysis and publication bias

The sensitivity analysis did not yield statistically significant results for all variables examined. Additionally, there was no indication of publication bias in studies evaluating the impact of MeD on BMI (P = 0.734 for Begg's test and P = 0.539 for Egger's test), and MFIS (P = 1.000 for Begg's test and P = 0.615 for Egger's test).

## 4 Discussion

In the present systematic review and meta-analysis comprising five RCTs, adherence to the MeD led to a reduction in BMI among patients with RRMS. Furthermore, a slight, yet statistically insignificant, decrease was noted in MFIS scores.

The MeD has been the subject of considerable interest about the potential impact on MS due to higher amounts of fruits, vegetables, whole grains, legumes, nuts, and olive oil, alongside a moderate intake of fish, and poultry. This dietary pattern is associated with reduced inflammation and oxidative stress, factors that are implicated in the pathogenesis of MS [24,25]. Furthermore, the high intake of omega-3 fatty acids from fish and olive oil, as well as the presence of polyphenols in fruits, may confer neuroprotective effects by modulating immune function and promoting antioxidative pathways [26]. Additionally, the limited intake of saturated and trans fats may contribute to improved overall health, which could benefit individuals with MS [27]. Notably, while evidence suggests that following a MeD can potentially ameliorate MS symptoms and reduce disease activity, further research is needed to elucidate the specific mechanisms underlying these effects and to establish definitive recommendations for individuals with MS. A systematic review published in 2023, reported that MeD is more recommendable than other diet models due to the positive health results reported in long-term studies and the absence of any side effects for MS patients [28].

The potential mechanisms and factors underlying the reduction of fatigue by the MeD in the context of MS involve various physiological processes and nutritional components. Firstly, the anti-inflammatory compounds found in fruits, vegetables, and olive oil may help dampen chronic inflammation, a major contributor to MS-related fatigue [29]. Additionally, the abundant antioxidants present in the MeD, particularly in fruits, vegetables, and olive oil, can mitigate oxidative stress, which is associated with fatigue and neurodegeneration in MS [30]. The inclusion of omega-3 fatty acids from fatty fish, known for their anti-inflammatory and potential neuroprotective effects, highlights another critical mechanism by which the diet may reduce neuroinflammation and subsequently alleviate fatigue in MS [31,32]. Furthermore, the modulation of gut microbiota induced by MeD influences psychological well-being and collectively suggests a multifaceted approach to alleviating MS-related fatigue [33,34].

In a scientific insight assessing the potential impact of the MeD on body weight in individuals with MS, it is essential to delve deeper into the underlying mechanisms and factors contributing to weight management. Logically, the dietary components and physiological effects of the MeD may influence body weight in the context of MS [35]. Firstly, the high fiber content of the diet, derived from whole grains, fruits, and vegetables, can be detailed for impact on promoting satiety and reducing overall energy intake, potentially supporting weight loss efforts [36]. Emphasizing the role of nutrient-dense foods and the presence of healthy fats, such as monounsaturated fats from olive oil and omega-3 fatty acids from fish, can be crucial in understanding how these components contribute to appetite regulation and metabolic processes related to weight management [37].

Further, the positive metabolic effects of the MeD, including insulin sensitivity, blood glucose regulation, and lipid metabolism, are integral to body weight regulation [38]. Additionally, the psychological aspects of the diet, such as sensory satisfaction from wholesome foods and the potential impact on eating behaviors, can be explored as contributing factors to weight management in individuals with MS [39].

Genetic studies have identified numerous genetic variants associated with obesity, including those involved in appetite regulation, energy metabolism, and adipose tissue function [40,41]. The MeD, which has potential to mitigate obesity and related metabolic disorders, may interact with obesity-related genetic predisposition to influence MS risk and progression [35,42]. The nutrient-rich, anti-inflammatory, and antioxidant properties of MeD may exert modulatory effects on pathways relevant to both obesity and MS, potentially influencing the interplay between genetic susceptibility, obesity, and MS [43,44]. Investigations into the differential effects of MeD on individuals with varying genetic predispositions to obesity and MS could provide valuable insights into personalized dietary recommendations for MS prevention and management.

In summary, MeD offers a multi-pronged approach to reducing fatigue and BMI improvement in individuals with MS. However, further research is necessary to fully elucidate these mechanisms and establish the causal relationships between MeD and clinical manifestations in MS. The present meta-analysis provides valuable insights for clinicians, researchers, and individuals with MS seeking dietary interventions for health promotion. Future health promotion interventions to promote MeD using evidence-based (theory-based) approaches are warranted.

### 4.1 Limitations

A limitation of the current meta-analysis was the absence of serologic and MRI data for all trials included, restricting the meta-analysis to only two variables (MFIS, BMI). Additionally, the limited number of RCTs precluded the feasibility of conducting sub-group analyses across the selected variables. To bolster the robustness of the findings, RCTs with larger sample sizes would be imperative for confirming the present results.

## 5 Conclusion

The current systematic review and meta-analysis of clinical trials unveiled that the Mediterranean diet holds promise in normalizing BMI in patients with RRMS. While a marginal and statistically insignificant decrease in fatigue severity was noted, additional trials are required to evaluate serological, functional, and physical parameters in the pooled analysis. Further investigation is warranted to better understand the efficacy of the Mediterranean diet in managing MS.

## Funding

The authors affirm that they have not received support from any commercial organization for the submitted study.

## Data availability statement

The datasets used and/or analyzed during the current study are available from the corresponding author, [Dr. Amir Reza Moravejolahkami; amimohs@gmail.com, moravejolahkami@gmail.com], on reasonable request.

## Code availability (software application or custom code)

Not applicable.

## Ethics approval and consent to participate

This systematic review was registered in the International Prospective Register for Systematic Review (PROSPERO) database (n. CRD42022368118; Reg. date: 2022.10.30).

## CRediT authorship contribution statement

**Amir Reza Moravejolahkami:** Writing – review & editing, Writing – original draft, Validation, Supervision, Software, Methodology, Investigation, Formal analysis, Conceptualization. **Mehdi Shakibaei:** Writing – review & editing. **Manoj Sharma:** Writing – review & editing. **Masoud Mohammadnezhad:** Writing – review & editing, Visualization, Resources, Project administration. **Sri Lakshmi Sravani Devarakonda:** Writing – review & editing.

## Declaration of competing interest

The authors declare that they have no known competing financial interests or personal relationships that could have appeared to influence the work reported in this paper.
