# Supplementary material for: Effect of Mediterranean diet on body mass index and fatigue severity in patients with multiple sclerosis: A systematic review and meta-analysis of clinical trials
Source: Heliyon. 2024 Sep 14;10(18):e37705. doi: 10.1016/j.heliyon.2024.e37705 (PMC11422037; doi:10.1016/j.heliyon.2024.e37705)
Supplement: Multimedia component 2 [file mmc2.docx]

**Manuscript title:** Effect of Mediterranean diet on body mass index and fatigue severity in patients with multiple sclerosis: a systematic review and meta-analysis of clinical trials

**Supplementary Table 2.** Search strategy details

| **Databases** | **Search terms** |
| --- | --- |
| **PubMed**  **(n=108)** | (((("Mediterranean Diet"[MeSH Terms] OR "MeD"[MeSH Terms] OR "Diet"[MeSH Terms] OR "Plant-based Diet"[MeSH Terms] OR "Healthy Diet"[MeSH Terms] OR "Mediterranean-like Diet"[tiab]))) AND (("Relapsing-Remitting Multiple Sclerosis"[MeSH Terms] OR "Multiple Sclerosis"[MeSH Terms] OR "RRMS"[tiab] OR "MS"[tiab])) AND (("Body Mass Index"[MeSH Terms] OR "Fatigue"[MeSH Terms] OR "Modified Fatigue Impact Scale"[MeSH Terms] OR "MFIS"[tiab] OR "BMI"[tiab])) AND (("Intervention"[tiab]) OR "Intervention Study"[tiab]) OR "Intervention Studies"[tiab]) OR "Controlled trial"[tiab]) OR "Randomised"[tiab]) OR "Randomized controlled trial"[tiab]) OR "Randomized clinical trial"[tiab]) OR "Randomized clinical trial"[tiab]) OR "RCT"[tiab]) OR "Non-Randomized Controlled Trials"[tiab]) OR "Clinical Trials as Topic"[MeSH Terms]) OR "Clinical Trials"[tiab]) OR "Clinical Trial"[tiab]) OR "Trial"[tiab]) OR "Trials"[tiab]) OR "Non-Randomized Controlled Trials "[MeSH Terms]) OR "Non-Randomized Controlled Trials "[tiab]) OR "Cross-Over Studies"[MeSH Terms]) OR "Cross-Over study"[MeSH Terms]) OR "Cross-Over study"[tiab]) OR "Cross-Over trial "[tiab]) OR "Cross Over trial "[tiab]) OR "Cross Over study"[tiab]) OR "Double-Blind Method"[MeSH Terms]) OR "Double-Blind Method"[tiab]) OR "Double-Blind "[tiab]) OR "Double-Blind trial "[tiab]) OR "Double-Blind study"[tiab])))) |
| **Scopus**  **(n=17)** | TITLE-ABS-KEY "Mediterranean Diet" OR "MeD" OR "Diet" OR "Plant-based Diet" OR "Healthy Diet" OR "Mediterranean-like Diet") AND TITLE-ABS-KEY ("Relapsing-Remitting Multiple Sclerosis" OR "Multiple Sclerosis" OR "RRMS" OR "MS") AND TITLE-ABS-KEY ("Intervention" OR "Intervention Study" OR "Intervention Studies" OR "Controlled trial" OR "Randomised" OR "Randomized controlled trial" OR "Randomized clinical trial" OR "Randomized clinical trial" OR "RCT" OR "Non-Randomized Controlled Trials" OR "Clinical Trials" OR "Clinical Trial" OR "Trial" OR "Trials" OR "Non-Randomized Controlled Trials " OR "Cross-Over study" OR "Cross-Over trial " OR "Cross Over trial " OR "Cross Over study" OR "Double-Blind Method" OR "Double-Blind " OR "Double-Blind trial " OR "Double-Blind study") |
| **Cochrane library**  **(n= 9)** | ("Mediterranean diet" AND "Multiple Sclerosis" AND "Trial") |
| **Embase**  **(n= 11)** | ("Mediterranean Diet" OR "MeD" OR "Diet" OR "Plant-based Diet" OR "Healthy Diet" OR "Mediterranean-like Diet") AND ("Relapsing-Remitting Multiple Sclerosis" OR "Multiple Sclerosis" OR "RRMS" OR "MS") AND ("Body Mass Index" OR "Fatigue" OR "Modified Fatigue Impact Scale" OR "MFIS" OR "BMI") AND ("Intervention" OR "Intervention Study" OR "Intervention Studies" OR "Controlled trial" OR "Randomised" OR "Randomized controlled trial" OR "Randomized clinical trial" OR "Randomized clinical trial" OR "RCT" OR "Non-Randomized Controlled Trials" OR "Clinical Trials" OR "Clinical Trial" OR "Trial" OR "Trials" OR "Non-Randomized Controlled Trials " OR "Cross-Over study" OR "Cross-Over trial" OR "Cross Over trial" OR "Cross Over study" OR "Double-Blind Method" OR "Double-Blind" OR "Double-Blind trial" OR "Double-Blind study") |
| **Google Scholar ***  **(n= 46)** | ("Mediterranean diet" AND "Multiple Sclerosis" AND "Trial") OR ("Plant-based Diet" AND "Multiple Sclerosis" AND "Trial") |
| **ScienceDirect**  **(n= 14)** | Title, abstract, keywords: ("Mediterranean diet" AND "Multiple Sclerosis" AND "Trial") |
| **ISI Web of Science**  **(n=23)** | (TS=(Mediterranean diet* AND Multiple Sclerosis AND Trial) OR TS=( Mediterranean diet* AND Relapsing-Remitting Multiple Sclerosis AND Trial) OR TS=(Plant-based Diet* Multiple Sclerosis AND Trial) OR TS=(Plant-based Diet* AND Relapsing-Remitting Multiple Sclerosis AND Trial)) AND LANGUAGE: (English) AND DOCUMENT TYPES: (Article)  *Indexes=SCI-EXPANDED, SSCI, A&HCI, CPCI-S, CPCI-SSH, BKCI-S, BKCI-SSH, ESCI Timespan=All years* |
| * just screened for the first ten page | |
